# Decision making under uncertainty in the diagnosis and management of Alzheimer's Disease in primary care: A study protocol applying concepts from neuroeconomics

**DOI:** 10.3389/fmed.2022.997277

**Published:** 2022-10-18

**Authors:** Gustavo Saposnik, Zahinoor Ismail, Anne-Marie Rivard, Debbie Knifton, Gillian Bromfield, Maria Terzaghi, Alonso Montoya, Marie-Chantal Menard

**Affiliations:** ^1^Division of Neurology, Department of Medicine, St. Michael's Hospital, University of Toronto, Toronto, ON, Canada; ^2^Decision Neuroscience Unit, Li Ka Shing Knowledge Institute, St. Michael's Hospital, University of Toronto, Toronto, ON, Canada; ^3^NeuroeconSolutions, Neuroeconsolutions.com, Toronto, ON, Canada; ^4^Departments of Psychiatry and Clinical Neurosciences, Hotchkiss Brain Institute, University of Calgary, Calgary, AB, Canada; ^5^Policy and Healthcare Ecosystem, Hoffmann-La Roche, Mississauga, ON, Canada; ^6^Medical Affairs Neuroscience, Hoffmann-La Roche, Mississauga, ON, Canada; ^7^Centre Praxis at Saint-Jean-sur-Richelieu, Saint-Jean-sur-Richelieu, QC, Canada

**Keywords:** Alzheimer's Disease, cognitive impairment, disease-modifying therapy, decision-making, inertia, risk, ambiguity, therapeutic inertia

## Abstract

**Background:**

The current management of patients with Dementia, primarily with Alzheimer's Disease (AD) is rapidly evolving. However, limited information is available about the current gaps and decision-making in primary care.

**Objectives:**

To evaluate factors associated with gaps, risk preferences regarding diagnostic and therapeutic choices in the management of patients with AD by primary care physicians (PCP) from across Canada.

**Methods:**

We propose a non-interventional, cross-sectional pilot study involving 120 primary care physicians referred from the College of Family Physicians of Canada to assess diagnostic and therapeutic decisions in the management of ten simulated AD-related case-scenarios commonly encountered in clinical practice. We initially describe the current landscape and gaps regarding diagnostic and therapeutic challenges in the management of patients with AD in primary care. Then, we provide concepts from behavioral economics and neuroeconomics applied to medical decision-making. Specifically, we include standardized tests to measure risk aversion, physicians' reactions to uncertainty, and questions related to risk preferences in different domains. Finally, we summarize the protocol to be implemented to address our goals. The primary study outcome is the proportion of participants that elect to defer initial investigations to the specialist and the associated factors. Secondary outcomes include the proportion of PCP willing to order cerebral spinal fluid studies, PET scans, or initiate treatment according to the simulated case-scenarios. The study will be conducted in English and French.

**Conclusions:**

The study findings will contribute a better understanding of relevant factors associated with diagnostic and therapeutic decisions of PCP in the management of AD, identifying participant's preferences and evaluating the role of behavioral aspects such tolerance to uncertainty, aversion to ambiguity, and therapeutic inertia.

## Introduction

Dementia is now the seventh leading cause of mortality globally and we know from the World Alzheimer's Report that it is the disease with the highest cost to society (1). Currently it is estimated that over 700,000 Canadians have dementia; by 2040 that number is expected to grow to over 1 million (2). Alzheimer's Disease (AD) is by far the most common type of dementia, accounting for 60–80% of all dementia diagnoses (3). Unfortunately, the majority of patients with dementia in Canada are diagnosed at later stages of the disease (1).

There has been significant scientific progress in the area of Alzheimer's Disease and a number of potential treatments are on the horizon, which hold promise in delaying progression of the disease. These treatments are most likely to be effective in people diagnosed at the early stages of disease. Novel diagnostic tests such as fluid-based biomarkers will be required alongside cognitive and behavioural testing in order to confirm Alzheimer's disease pathology in people in the early stages of disease. At present these tests are not currently recommended for routine use (2). This enhanced diagnosis process will be critical for identifying potential patients most likely to benefit from disease-modifying treatments.

With the impending therapeutics breakthrough, accurate disease diagnosis will be required to access treatments. Accordingly, the demand for new biomarkers and imaging technology will increase. Up until now, those investigation tools were dedicated primarily to research, with little guidance on clinical use. Many family physicians may defer utilization of these novel investigations to specialists. However, it will take collaboration amongst several health care providers to accomplish the heavy task of caring for this growing group of patients.

Several questions remain unanswered regarding the changing landscape of dementia care in Canada. What is the knowledge base and comfort level of primary care practitioners (PCPs) in Canada to investigate and manage neurocognitive disorders, especially with these emerging therapies? Would PCPs be willing and able to perform all the tests required to ascertain whether a person has dementia or not, and which specific dementia? Will they be comfortable enough to start a treatment? How do regional differences in practice styles, access to investigations, and reimbursement and coverage for medications influence a PCPs approach?

The goals of this study protocol are to: (i) answer these questions regarding the willingness of PCP to initiate diagnostic and treatment decisions in management of AD, (ii) provide an overview of the existing gaps in delivery of care for patients with AD, and (iii) provide the rationale and summary of the proposed study applying concepts from behavioral economics and neuroeconomics.

## Current landscape of the diagnosis and management of AD in primary care

Notwithstanding the longstanding consensus in Canada that that timely detection, diagnosis, and care of those living with dementia is mainly the responsibility of PCPs (4), there is uncertainty around the impact on quality of care, and the willingness and/or preparedness of PCPs to take on this role (5). Specifically, a position paper developed in conjunction with the College of Family Physicians of Canada's Health Care of the Elderly Program Committee describes family physician roles in key aspects of dementia care including: (1) contributing to dementia prevention, (2) providing a timely diagnosis, (3) excluding other conditions that might look like dementia, (4) determining the stage of dementia, (5) communicating a diagnosis of dementia with dignity, and (6) providing postdiagnosis management and person-centred, integrated care (4). A survey of both primary care and specialist physicians found that confidence amongst primary care physicians in the ability to provide dementia care has improved in the last decade, but several issues warrant specialist involvement including management of behavioral and psychological symptoms of dementia (i.e., neuropsychiatric symptoms), diagnosis of atypical dementias, and management of complex co-morbid conditions involving polypharmacy. Addressing driving safety, in addition, was an area that family physicians felt uncomfortable dealing with, with the desire for specialists to make potential rapport-impairing decisions with respect to driving. Further, physicians felt that community supports were confusing and difficult to access, inter-individual and inter-practice differences were significant in terms of communication between family physicians and specialists, and shared and collaborative care might optimize delivery of dementia care (6).

Strategies and interdisciplinary models of primary care to address the issue of timely diagnosis vary by province (7–9). Some of these models that can help to address the issue of timely diagnosis include MINT Memory Clinics and family health teams (ON) (https://www.hqontario.ca/Quality-Improvement/Quality-Improvement-in-Action/ARTIC/ARTIC-Projects/Primary-Care-Collaborative-Memory-Clinics), the Rural and Remote Memory Care Clinic (RRMC) in Saskatchewan (https://cchsa-ccssma.usask.ca/ruraldementiacare/) (10), family medicine groups (QC), and Primary Care Networks (AB). Recent findings from the Quebec Alzheimer Plan (8) demonstrated that PCPs attitudes toward providing dementia care are positive and willing, but that they would benefit from more support and training. Furthermore, recent research to better understand how to address these gaps found that higher levels of institutional support such as financial support, training, and multi-disciplinary teams were strongly associated with a higher quality of dementia care in primary care practices (5). However, these innovative models of care do not exist everywhere in Canada, so the challenge of how to reduce barriers to care at the primary care level remains in many other areas.

Quality of dementia care in primary practice has been associated with higher degrees of institutional support (e.g., financial and training support and interdisciplinary teams) (5). Evidence has also explored post-diagnostic dementia care in primary care settings and has suggested that a range of primary care-led dementia care models may be acceptable and feasible. To improve quality of care, it was suggested that services should add dementia-focused health professionals into primary care, develop primary care leadership in dementia, provide sufficient funding and collaboration opportunities, and ensure community service links and social support are included (11). Although experiences vary by jurisdiction, one study of relatives and caregivers of persons with dementia in Manitoba faced challenges when accessing primary, specialist, and home healthcare services. These participants recommended providing more doctors, being aware of available resources, addressing health issues in a timely fashion, increasing the number of health professionals with dementia-specific knowledge, providing more information early in the disease course, and creating more capacity in personal care homes. Additionally, it was emphasized that families have relevant information about the person with dementia, and throughout the continuity of care, there should be effective collaboration between family doctors, family caregivers, and other health professionals (12).

A recent scoping review and environmental scan is informative about primary care dementia services in Canada. This study identified 18 different primary care-based models of dementia care for persons in the early and middle stages of dementia. However, despite 5 of Canada's provinces and territories having dementia strategies (British Columbia, Alberta, Manitoba, Quebec and Nova Scotia), only 2 primary care memory clinic models have been implemented in Canada (9). The Primary Care Collaborative Memory Clinics (PCCMC) service delivery model integrates interprofessional healthcare providers into primary care settings, and partners with community agencies to meet the multifaceted needs of the dementia population. Initial evaluations have suggested that the PCCMC model is generalizable to multiple family practice settings, but challenged by lack of sustainable funding, inadequate infrastructure support, managing competing priorities, maintaining adequate communication among team members, and coordinating multiple schedules. Relatedly, Multispecialty Interprofessional Team (MINT) memory clinics have been explored for provision of dementia services in primary care. Surveys of staff working in these clinics found that this approach was associated with lower perceived challenges in provision of care and greater enthusiasm for this type of work (10). The emergence of these memory clinics has increased the profile of primary care clinics in diagnosis and management of dementia.

Another model, although not dementia-specific, is in use in Alberta. Specialist LINK is a real-time, non-urgent telephone collaboration line designed to link family doctors and specialists, in which the standard of care is for specialists to return the primary care physician's call within 1 h. One of the goals is to improve efficiency and enhance the coordination of patient care through enhanced communication between primary and specialty care, addressing a limitation raised in many studies of dementia care in primary care settings (13). Although performance metrics are not yet available, this approach can potentially improve both diagnosis and management of patients with dementia.

Dementia guidance is also relevant to the primary care role in dementia care. Since 1989, the Canadian Consensus Conference on the Diagnosis and Treatment of Dementia (CCCDTD) has been generating clinical guidelines for dementia care, with the 5^th^ iteration published in 2020 (2). Eight topics were addressed including: (1) utility of the National Institute on Aging research framework for clinical Alzheimer's disease (AD) diagnosis (14); (2) updating diagnostic criteria for vascular cognitive impairment, and its management (15); (3) dementia case finding and detection (16, 17); (4) neuroimaging and fluid biomarkers in diagnosis (18); (5) use of non-cognitive markers of dementia for better dementia detection (19); (6) risk reduction/prevention (20); (7) psychosocial and non-pharmacological interventions (21); and (8) deprescription of medications used to treat dementia (22). Many of these themes and recommendations can be implemented primary care. However, with the potential emergence of disease-modifying drugs traditional approaches to dementia detection in primary care may be less effective at identifying early-stage dementia. Specifically, at the time of writing, incorporating the National Institute of Aging biological definition and clinical staging for AD was relegated to research rather than clinical care (14). But, if amyloid positivity is required for use of disease-modifying drugs, there exist significant roadblocks in primary care to this entry criterion for drug use, whether it be determination of amyloid status with fluid biomarkers or with PET tracers. Thus, additional exploration of barriers and solutions to effective detection and management of dementia in primary care is required. The proposed study, informed by principles of neuroeconomics, may further this goal.

## Understanding our ecosystem: Identifying gaps in the diagnosis and management of AD

In Canada, strategies and guidelines to support diagnosis and management of people with dementia are rooted in primary care (2, 19, 23–25). However, it is welldocumented that family physicians report feeling poorly equipped to diagnose and provide care for older adults living with dementia (26). Furthermore, only 2 out of 5 Canadian doctors feel wellprepared to manage dementia care in a community setting (6, 27). It is not surprising that the diagnosis of dementia occur at later stages of disease (28).

The case-scenario illustrates some of the current challenges. For example, physicians who are in the front line (e.g., PCPs), may not be able to order or have the expertise to interpret the results of more sophisticated tests (PET or SPECT scans, biomarkers) to confirm the diagnosis of AD, whereas specialists may not have a close contact to patients and their families and caregivers to monitor the treatment and behavioral complications. Given the initial undifferentiated clinical presentation of many patients with Dementia and the slowly evolving underlying pathology, delays in the diagnostic are expected. Altogether these factors alter the efficient management of patients with AD.

There are a number of reasons for a delay in timely diagnosis of cognitive impairment or dementia: stigma against dementia amongst the general public is one of them (29, 30), and also well documented amongst family physicians. In a recent survey conducted for Alzheimer's Disease International, 33% of clinicians surveyed believed that nothing can be done about dementia. Some of these negative views can impede diagnosis including a perceived lack of therapeutic benefits of early diagnosis, the feeling that nothing can be done for the patient, and an unwillingness to communicate a dementia diagnosis (26). PCPs also feel that their training has been insufficient to support diagnosis particularly in the earlier stages of the disease.

**Figure 1:**
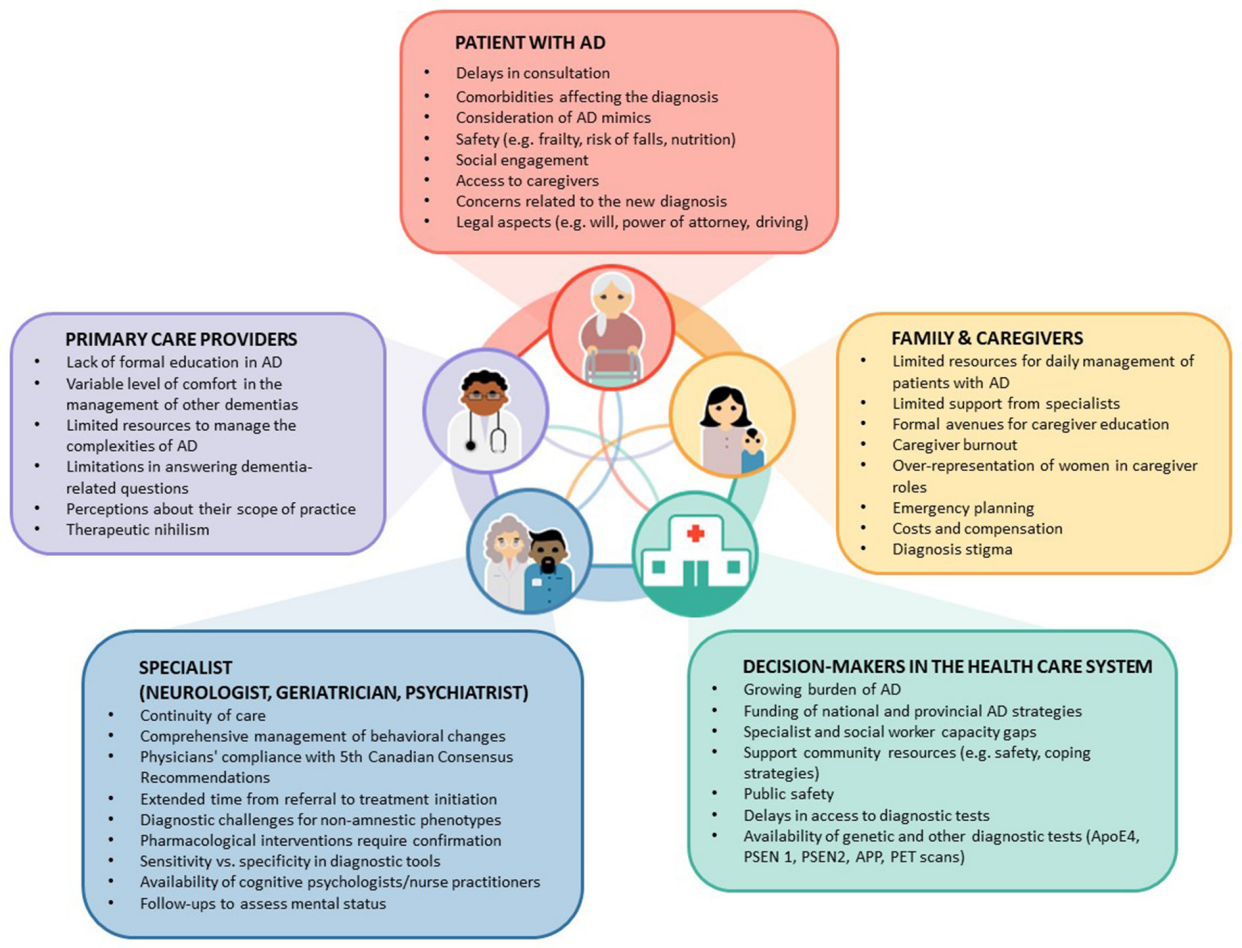
Gaps and barriers in the diagnosis and management of patients with Alzheimer's Disease (AD) in primary care. This figure illustrates factors and existing gaps in the management of patients with AD. It shows the complex interactions among key players: Patients, family members and caregivers, and decision-makers. Text in the boxes identify specific gaps or issues. Note that the most common point to initiate investigations starts by symptoms disclosed by patients or their family members and caregivers.

Figure 1 illustrates additional barriers, including time and financial constraints, limited resources for diagnostic work up at point of care, diagnostic uncertainty, and gaps in knowledge and skills for making a differential diagnosis (28). Perhaps, one of the major health care gaps is the lack of integrated solutions to improve dementia care.

The growing body of evidence around the positive impact of dementia risk reduction strategies (31), the advent of new diagnostic tools, and the likelihood of disease-modifying treatments being indicated for use in the early stages of Alzheimer's Disease all point toward the important role that primary care plays as “dementia first responders” now and in the future. There is a need to better understand specific factors that impact PCPs readiness and ability to manage dementia effectively, and their decision-making pathways when faced with a patient exhibiting new cognitive or behavioral concerns. Our study will complement the recent body of knowledge around barriers to timely diagnosis and will help support the development of programs and strategies to address these gaps.

Box 1 **Illustrative Case**

A 71-year-old woman presented for evaluation for 18-month history of progressive memory loss. Her husband reported that she forgets passwords and sometimes repeats the same questions. In the last 6 months she had trouble completing some routine tasks (e.g., cooking, grocery shopping). She was otherwise independent with day-to-day function. She has seen her PCP (e.g., family physician [FP]) 8 months ago, but she did not raise any concerns regarding her mental status. Her past medical history includes hypertension, but otherwise was unremarkable. Review of medications did not reveal any agents known to affect cognition. Her physical exam was unremarkable. On cognitive testing, she scored 23/30 on the Mini Mental State Examination (MMSE) and 22/30 in the Montreal Cognitive Assessment (MoCA), losing points for orientation, word recall, and serial 7s. She showed mild deficits on tests of executive, language, and visuospatial functions. Her FP ordered blood tests and a CT scan of the head. Laboratory evaluations for reversible causes of cognitive impairment were within normal limits. A CT scan of the head showed atrophy of the hippocampus and medial temporal lobes bilaterally, with no significant vascular lesions. Due to a minimum 6-month waiting time to see a specialist, the FP thought about ordering additional investigations (e.g., PET scan, CSF studies, biomarkers), but these were only available to specialists. By the time of the next follow-up appointment, after completion of blood work and CT head and reassessment, four more months had elapsed. The patient, family members, and caregivers expressed concerns about disease progression and felt overwhelmed when informed of the presumed diagnosis. Initial therapeutic options and potential side effects were discussed, additional tests to monitor the treatment, and follow-up every 4 to 6 months was offered. Ultimately, a referral to a specialist was made.

## Applying concepts from neuroeconomics in the management of patients with AD

Neuroeconomics is the science that studies the principles of how we make decisions (32, 33). It is based on concepts from behavioral economics, advanced brain imaging and electrophysiological studies (e.g., magnetoencephalography, skin conductance test, pupillary variability as a marker of central arousal) and the application of mathematical algorithms (34, 35).

In behavioral economics, uncertainty is a general term that comprises two concepts: risk and ambiguity. Risk applies to events with known probability, whereas ambiguity is a term reserved for events for which probabilities are unknown. Uncertainty is one of the most important contributing factors affecting decisions in medical care (33). Making diagnostic or therapeutic decisions is a complex task as it requires examining risks and comparing alternative options with incomplete information under uncertainty. For example, most commonly clinicians initiate treatments (e.g., hypertension, mood disorders, dementia, migraine, etc.) without knowing how specific genes may alter the metabolism of a drug or the risk of serious side effects, ultimately affecting the therapeutic response (pharmacogenetics) (36). Decisions based on erroneous assessments may result in suboptimal medical care and outcomes, as well as unmet needs for patients and their families. Several reports have highlighted examples of care gaps or suboptimal management of patients with or suspected of AD (e.g., lack of treatment, mismanagement of behavioral changes, limited family and caregiver support) (2, 37–39).

Diagnostic and therapeutic decisions in the management of AD depend on patient, clinician and health system factors (Figure 1). As mentioned, despite the continuous advances in the management of AD, we need a better understanding of patient, family member, and caregiver preferences to overcome the existing gaps for the implementation of shared-decision making under the paradigm of personalized medicine.

Two theories are relevant to medical decisions in the management of patients with dementia: (1) The Dual Process Theory (DPT) and the Prospect theory (PT). The DPT suggests that human decisions are commanded by two hierarchical processes, described as System 1 (intuitive) and System 2 (analytical). In brief, System 1 refers to an automatic, unconscious, fast, and effortless (or routine) mechanism to make most common decisions (e.g., from buying the usual milk in a grocery store to starting an antihypertensive agent in patients naïve to treatment and without relevant comorbidities). Conversely, System 2 makes deliberate decisions that require a more thoughtful process, usually slower than System 1 and conscious (40) (e.g., going to a new grocery store that carries unfamiliar milk brands or the selection of an antihypertensive drug in someone with secondary hyperaldosteronism). Although the DPT has been questioned, it provides a simple framework to understand medical decisions (see details at https://bit.ly/2R3p9Ox). In practical terms, clinicians make automatic diagnostic choices when facing a consultation about cognitive impairment (e.g., ordering blood test to identify common factors associated with a reversible cognitive impairment and brain imaging are automatic decisions—System 1). Conversely, the detection of more complex clinical scenarios from the medical history, physical and cognitive assessment (e.g., clinical course, family history of AD, affected domains of cognition) may require a more thoughtful process -System 2- to determine use of additional investigations (e.g., biomarkers, PET scan, cerebral spinal fluid-CSF). We hypothesized that participants will make automatic decisions (involving system 1) for straightforward simulated scenarios where there is limited degree of uncertainty, compared to deliberate choices when face more complex scenarios (system 2).

The Prospect Theory (PT) is a behavioral model that explains decisions between alternatives that involve risk and uncertainty (e.g., % likelihood of gains or losses) (41). For example, PT shows that people think in terms of expected utility (benefit) relative to a reference point (e.g., current wealth) rather than absolute outcomes (41). Although PT was developed under the economic framework, it has wider applications. In medicine, PT suggests that both a patient's acceptance of a treatment and a physician's therapeutic recommendations would change depending on the utility function (perception of net benefit) compared to their current health status (42). For example, patients with mild or no symptoms having a low risk of developing a disease progression or a serious medical conditions may elect to avoid “risky” treatments (given the low gains while having a significant risk of developing side effects), whereas patients with symptoms interfering with their quality of living or being at high risk of developing a disease or a medical complication would be willing to accept more risky treatments (given the higher gains even if the likelihood of side effects is increased).

Regarding practical management of patients with dementia—suspected of having AD—, these theories suggest that clinicians must weigh the risks and potential gains of a procedure or treatment, but also gather information regarding the “reference point” of an individual patient and their family members. New and more invasive procedures (e.g., spinal tap for the detection of biomarkers of AD—Aβ42, phosphorylated tau, and total tau—in the CSF) are now available to improve diagnostic accuracy, but not necessarily incorporated in the routine clinical practice or reimbursed appropriately (18, 43, 44). Similarly, new treatments (e.g., aducanumab, combination therapies with acetylcholine enhancers) are becoming available for specific patient selection, but required greater commitment than previous treatments (19, 45). We hypothesized that participants would not be willing to request biomarkers, specialized (SPECT) or invasive tests (spinal tap for CSF analysis) in the current landscape. In summary, clinicians have the difficult task to have up-to-date medical information and apply tools to make simple automatic decisions and more complex ones that aligns with patient and family goals and preferences.

## Summary of proposed study

**Figure 2:**
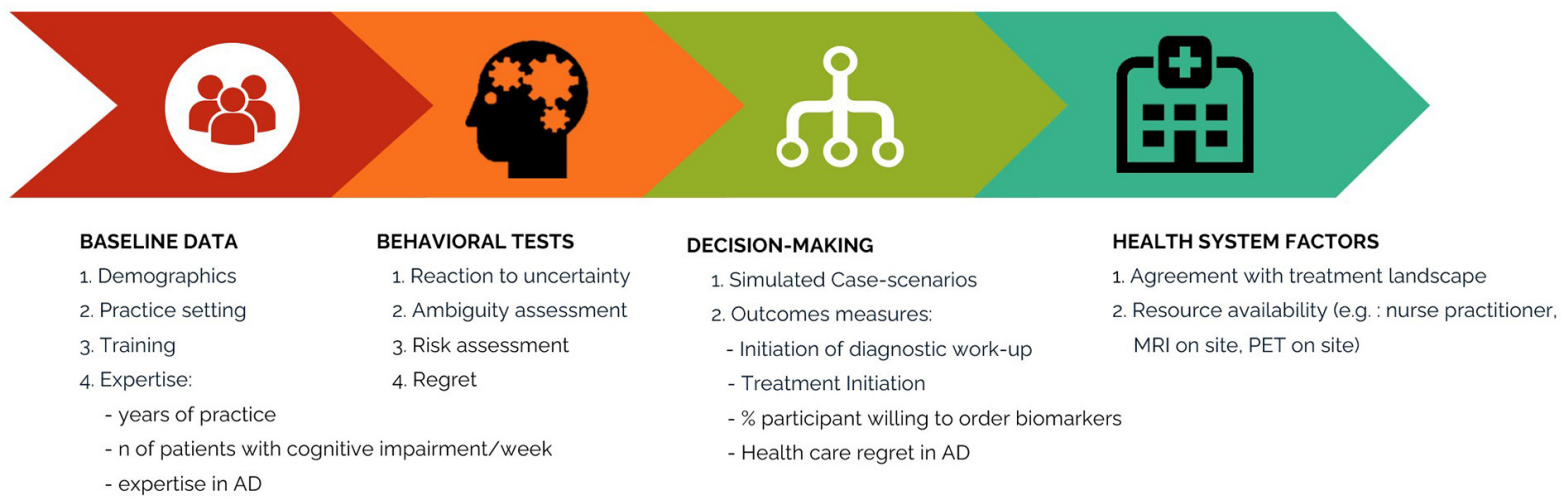
Flow of the proposed study. This figure illustrates the study flow. Participants start by completing information on demographics, medical education, and clinical experience, then behavioral tests to determine their risk preferences and subsequently 10 simulated case-scenarios related to common clinical encounters in the diagnosis and management of patients with dementia and AD. Finally, they will answer questions related to their current practice and resources.

Building on our previous work, we apply principles from neuroeconomics and behavioral economics to therapeutic decisions (42, 46, 47). The overarching goals of the proposed study are to: (i) evaluate factors associated with gaps in the care of patients with AD by PCPs across Canada, (ii) assess PCP risk preferences regarding diagnostic and therapeutic alternatives in the management of patients with AD, and (iii) explore health-care related regret (Figure 2, Study flow). This protocol constitutes the initial step to understand the current factors influencing the diagnosis and management of AD in primary care. Our results will serve to create and subsequently assess an educational intervention in a randomized study as previously done by our team (35, 48).

### Experimental design

This research is an online, non-interventional study starting in April 2022, anticipating completion by September 2022. The study will be conducted according to the ethical principles of the Declaration of Helsinki, and in compliance with the study protocol, International Conference on Harmonisation-Good Clinical Practice regulations, and applicable clinical trial regulatory requirements of Health Canada. Approval will be obtained from the research ethics board. Online informed consent will be obtained from all participating physicians.

### Study participants

We will enroll 120 PCPs caring for patients with Alzheimer's disease (AD) from across Canada. A PCP is a specialist in family medicine who provides definitive care to the undifferentiated patient at the point of first contact and takes continuing responsibility for providing the patient's comprehensive care (49). Participant selection will include referrals from the College of Family Physicians in Ontario, Quebec and British Columbia representative of the three largest Canadian provinces. The recruitment strategy is competitive on a first come first served basis until reaching a 40-participant quota from each of the three provinces.

### Study implementation

Participants will provide demographic information, practice settings and risk preferences according to our previous studies (42, 46). Subsequently, they will be exposed to ten simulated case scenarios to select a diagnostic or therapeutic choice. Finally, we will assess participants risk preferences, tolerance to uncertainty, and health-care regret (see description below).

## Participant risk preference, tolerance to uncertainty, and health care related regret

**Figure 3:**
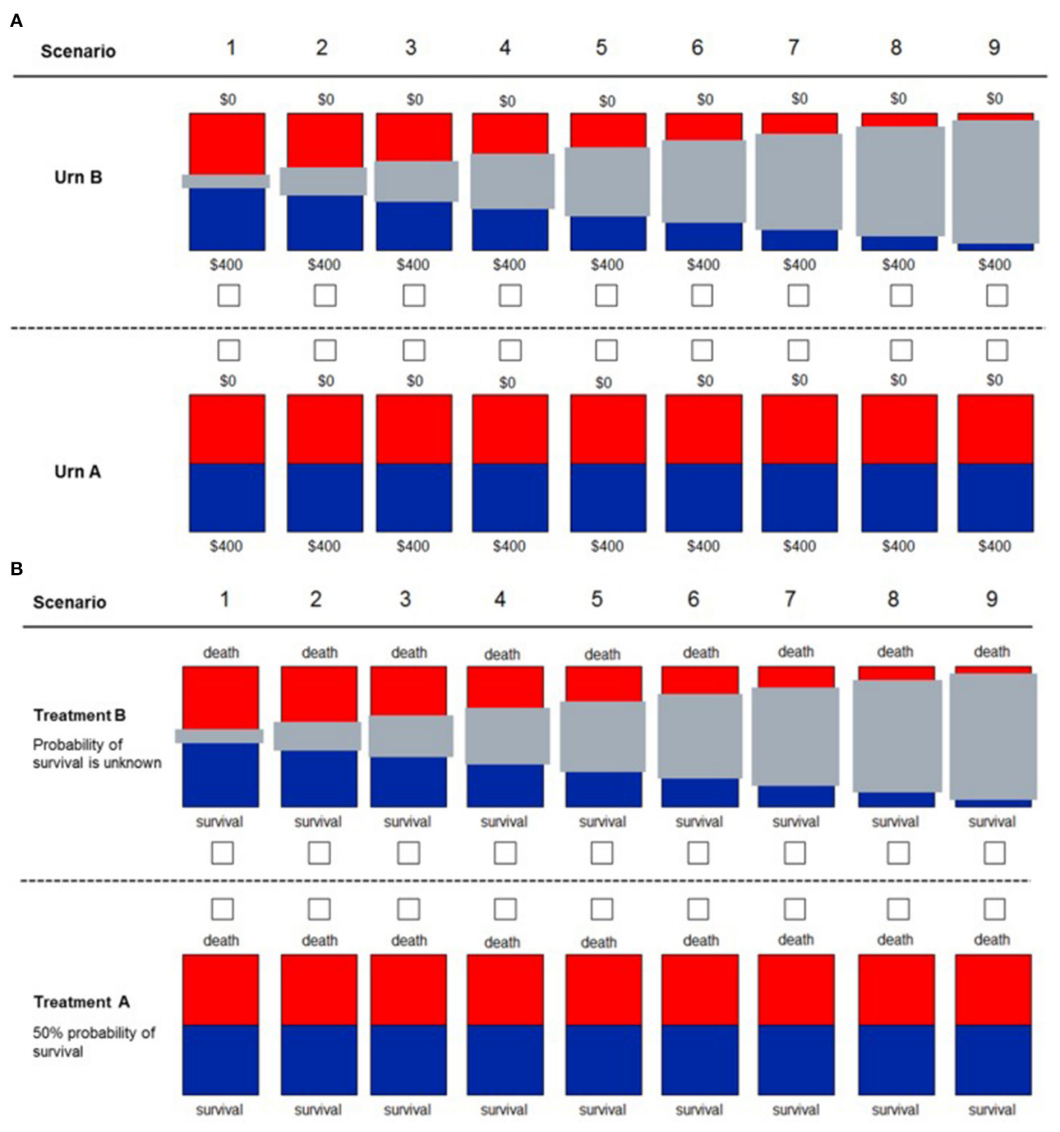
Decision scenarios used to measure ambiguity in financial **(A)** and health **(B)** domains. Participants are presented with two different urns. For urn type A, they knew that 50% of the balls were red and the other 50% were blue. For urn type B, they do not know the exact proportion of blue to red balls, with the gray bar representing the unknown proportion of balls (degree of uncertainty). For the financial domain, participants know that if they drew a blue ball, they would win the full amount of $400. If they drew a red ball, they would win $0. For the health domain, participants have to decide between two treatments for a patient. With “Treatment A,” the patient had a 50% probability of survival. With “Treatment B,” the exact probability of survival was unknown, with the gray bar representing the unknown probability (uncertainty). This figure was reproduced from Saposnik et al. (46).

As in previous studies, we designed behavioral experiments to assess participant risk preferences and tolerance to uncertainty (46). In brief, participants will be asked to choose between two options: either winning CAD$ 400 (equivalent to USD 315) or $0 when the probability is 50/50 (represented by a blue/red bar) vs. an option of unknown probability (represented by a blue/red bar covered by a gray bar) of the same outcome (Figure 3). Participants who favor the known probability of 50/50 are considered to have aversion to ambiguity. Risk will be assessed by asking participants to provide the minimal dollar amount that they would prefer over a 50/50 chance of winning CAD$400. The degree of risk aversion of each individual corresponds to the difference of the expected value of the risky option (CAD$ 200) minus the participant's response (50). Physician tolerance to uncertainty in a patient's care will be assessed by using the reaction to uncertainty test (51). The test comprises five questions that the respondent rates from 0 to 5, which are added to give a total score. Higher scores represent lower tolerance to uncertainty. Low tolerance to uncertainty is defined as values above the median of the total score. Further details of the protocol are published elsewhere (42). Regret will be assessed by the decision regret scale (DRS) (52). The DRS is a validated scale in which participants score five statements regarding regret on recent decisions in the diagnosis or treatment of patients with AD. High scores represent high level of regret (52).

Simulated case-scenarios have been created by our team of experts in the field, including PCPs with interest in medical education and AD (AF, MCM), a geriatric neuropsychiatrist (ZI), and two neurologists (AM, GS), one of them with expertise in online studies and decisions neuroscience (GS). All case-scenarios represent common medical encounters in the management of patients with cognitive impairment (see Appendix). The estimated total time of study completion per participant is 30–35 min.

### Outcome measures

The primary outcome of the study is the proportion of participants that elect to defer initial investigations to the specialist. Secondary outcomes include the proportion of PCP that are willing to order CSF study, a PET scan, or initiate DMTs according to the simulated case-scenarios.

### Analysis

The planned analysis includes generating descriptive demographics, variables of interest and decisional outcomes. Multivariable logistic regression and mixed effects models accounting for clustering analysis adjusting for age, sex, years of expertise, and health care-related regret will be completed (52).

## Expected results and future directions

This project will build upon our previous studies applying novel concepts from behavioral economics and Neuroeconomics (35, 42, 46–48, 53–56) and advance our knowledge on: (1) factors associated with practice gaps in the management of patients with dementia by PCPs across Canada; and (2) how the introduction of a DMT would modify treatment gaps. For example, we expect to estimate the the frequency of treatment initiation for case-scenarios with mild, moderate and advance AD, the use CSF diagnostic tests and new therapies (e.g., aducanumab) by PCPs. Our study introduces an innovative approach in medical education by applying concepts from Behavioral Economics and Neuroeconomics to AD care. For example, this innovative approach may help overcome the barriers to intensify treatment in AD care. Further, it may increase awareness and cognitive reflection in participants regarding effective diagnostic and therapeutic options in AD care, that would result in better clinical outcomes and patient quality of life. Main results are expected to be published and will serve as the background for further similar studies among general neurologists and development of educational interventions to optimize the early diagnosis and management of AD (57).

In conclusion, our study will answer questions regarding PCPs willingness to initiate diagnostic and therapeutic decisions in management of AD, identify the association between family physician's risk preferences and tolerance to uncertainty in diagnostic and treatment choices. Our results will provide background information to develop educational interventions to assist PCPs in improving the management and optimizing delivery of care for patients with AD.

## Ethics statement

Ethical approval was not provided for this study on human participants because Ethical approval was waived. The patients/participants provided their written informed consent to participate in this study.

## Author contributions

GS conceived the study, designed the protocol, created the simulated case-scenarios and the behavioral battery, and drafted the manuscript. MT participated in the design of the study including the behavioral battery and made intellectual in drafting the manuscript. A-MR, DK, GB, and M-CM participated in the design of the study and made intellectual in drafting the manuscript. ZI actively collaborated with GS in reviewing the case-scenarios and co-drafted the manuscript.

## Funding

This study was supported in part by NeuroEconSolutions and funded by an unrestricted grant from Roche Canada. Neither the sponsors nor Roche were involved in the design of the study. The preparation of this manuscript and decision to submit was the sole intention of the authors.

## Conflict of interest

Author GS was supported by a peer-reviewed career award from the Heart and Stroke Foundation of Canada Career Award. Authors AM, A-MR, GB, and DK were employed by Roche. Author ZI has received honoraria from Lundbeck/Otsuka outside the submitted work. The remaining authors declare that the research was conducted in the absence of any commercial or financial relationships that could be construed as a potential conflict of interest.

## Publisher's note

All claims expressed in this article are solely those of the authors and do not necessarily represent those of their affiliated organizations, or those of the publisher, the editors and the reviewers. Any product that may be evaluated in this article, or claim that may be made by its manufacturer, is not guaranteed or endorsed by the publisher.
