# Supplementary material for: Decision making under uncertainty in the diagnosis and management of Alzheimer's Disease in primary care: A study protocol applying concepts from neuroeconomics
Source: Front Med (Lausanne). 2022 Oct 18;9:997277. doi: 10.3389/fmed.2022.997277 (PMC9623110; doi:10.3389/fmed.2022.997277)
Supplement: Supplementary file 1 [file Data_Sheet_1.pdf]

SIMULATED CASE-SCENARIOS

1. One of your patients, an 85-year-old woman, was admitted to hospital for heart failure 2 weeks ago. She has a past history of hypertension. She is now back in your clinic. You have not seen her in the last 12 months. She developed delirium on day 2 of her hospital stay and was agitated, trying to pull out her IV, and striking out at the nurses. She received Quetiapine 25 mg BID for delirium, which resolved gradually over the following week. The family mentioned that she started having some confusion and forgetfulness in the last 6 months. Her physical exam is non-contributory.

What would you do? Please select <u>one option from the screen</u>:

- Complete blood-work, a cognitive assessment, and brain CT (or MRI) imaging.
- Given the history, you complete a cognitive assessment during the same visit and consider initiating treatment if below the normal range.
- Order brain imaging before proceeding with further investigations.
- Refer to a geriatrician or a general neurologist (Waiting time 3-4 months)
- Refer to another specialist (e.g. geriatric-psychiatrist, behavioral neurologist) (waiting time 6-9 months)

2. A 75-year-old man was referred to your practice from a retiring colleague regarding memory loss. The patient's daughter described that he has been more confused, forgets conversations, and can't remember where he put things, over the last year or two. There is no history of hallucinations, tremors, or other neurological symptoms. However, other than remembering passwords and home banking, he independent for his ADLs and IADLs. He has history of hypertension and dyslipidemia. General physical examination was normal. He was able to recite the months of the years backwards from December, but he omitted August. His MMSE score was 23/30. Blood work and urine culture are normal. A CT head completed 6 months ago showed no bleeds, hydrocephalus, or ischemic strokes. He was told by your colleague that this could be normal aging vs. mild cognitive impairment.

What would you do? Please select <u>one option from the screen</u>:

- Complete an MRI head and reassess
- Initiate treatment with an acetylcholinesterase (AChE) inhibitor and reassess
- Initiate treatment with an acetylcholinesterase (AChE) inhibitor and Memantine and reassess
- Refer to a geriatrician or a general neurologist (Waiting time 3-4 months)
- Refer to another specialist (e.g. geriatric-psychiatrist, behavioral neurologist) (waiting time 6-9 months)
- This patient needs a follow-up to determine the clinical course, and assessments of cognition and function (IADLs) to determine if treatment is warranted.

3. A 72-year-old woman presented for evaluation for a 2-year history of progressive memory loss. Her husband reported that she forgot passwords and repeated the same questions. She had trouble completing some routine tasks (e.g., cooking, grocery shopping). She was otherwise independent with day-to-day function. Her past medical history includes hypertension, diabetes mellitus, and hyperlipidemia. Review of medications did not reveal any substances known to affect cognition. Family history was notable for late-onset dementia in her mother and maternal grandmother.

Her physical exam is unremarkable. On cognitive testing, she scored 22/30 on the Mini Mental State Examination (MMSE)(and same value using MoCA), losing points for orientation, word recall, and serial 7s. She showed mild deficits on tests of executive, language, and visuospatial functions. Laboratory evaluations for reversible causes of cognitive impairment were within normal limits. Brain MRI showed atrophy of the hippocampus and medial temporal lobes bilaterally, with no significant vascular lesions.

What would you do? Please select <u>one option from the screen</u>:

- Initiate treatment with a acetylcholinesterase (AChE) inhibitor and reassess
- Initiate treatment with a acetylcholinesterase (AChE) inhibitor and Memantine and reassess
- Order Amyloid β and Tau biomarker prior to initiating treatment
- Refer to a geriatrician or general neurologist (Waiting time 3-4 months)
- Order biomarker tests of amyloid and tau prior to sending a referral to a neurologist
- Refer to another specialist (e.g. geriatric-psychiatrist, behavioral neurologist) (waiting time 6-9 months)
- This patient needs a follow-up to repeat the cognitive and functional assessment before deciding if treatment is warranted

4. A 74-year-old teacher man presented for evaluation of 1-year history of being more distractive. His wife reported that he misplaces personal items, and forgets proper names. There are no other significant deficits in ADLs or IADLs. He denies feeling depressed or having insomnia. His past medical history includes hypertension. He is taking perindopril 8 mg po od and Vit D 2000 units. There is no family history of neurological conditions. His physical exam is unremarkable. Administration of the MMSE (27/30) and MoCA (26/30) losing 3 points for word recall and 1 point for fluency.

What would you do? Please select <u>one option from the screen</u>:

- Order brain CT (or MRI) imaging and reassess
- Order blood work to rule out reversible causes of cognitive impairment and reassess
- Order Amyloid β and Tau biomarkers and reassess
- A and B and reassess
- A, B and C and reassess
- Refer to a geriatrician or general neurologist (Waiting time 3-4 months)
- Refer to another specialist (e.g. geriatric-psychiatrist, behavioral neurologist) (waiting time 6-9 months)
- This patient needs a follow-up to repeat the cognitive assessment before deciding if a referral or treatment are warranted

5. A 65-year-old woman presented with three years of declining physical activity and failure to complete work tasks. Her boss described her becoming 'lazy' and often just sitting at her desk. Her husband described her as unable to "think properly", sometimes resulting in wrong decisions or poor judgment. There has been no inappropriate behavior or depression. On the Mini-Mental State Examination (MMSE), she scored 28/30. She also had a reduction in his initiative, became quieter and spends more time sitting. Her MoCA score was 27/30. She generated 9 words beginning with the letter *F* in 1 minute. Memory testing showed impairment in delayed recall. The rest of the neurological exam was normal.

What would you do? Please select <u>one option from the screen</u>:

- Order brain CT (or MRI) imaging and reassess
- Order blood work to rule out reversible causes of cognitive impairment and reassess
- Order Amyloid β and Tau biomarkers and reassess
- A and B, and reassess
- A, B and C, and reassess
- Refer to a geriatrician or a general neurologist (Waiting time 3-4 months)
- Refer to another specialist (e.g. geriatric-psychiatrist, behavioral neurologist) (waiting time 6-9 months)
- This patient needs a follow-up to repeat the cognitive assessment before deciding if a referral or treatment are warranted

Follow up question (SAME CASE)—CANNOT GO BACK TO THE SCREEN

5ii. An MRI head showed more frontal than parietal atrophy. Blood work was non-contributory.

What would you do? Please select <u>one option from the screen</u>:

- Order Amyloid β and Tau biomarkers and send a referral to a neurologist
- Refer to a geriatrician or a general neurologist (Waiting time 3-4 months)
- Refer to another specialist (e.g. geriatric-psychiatrist, behavioral neurologist) (waiting time 6-9 months)
- This patient needs a follow-up to repeat the cognitive assessment before deciding if a referral or treatment are warranted
- Initiate treatment with acetylcholinesterase (AChE) inhibitors and reassess
- Initiate treatment with acetylcholinesterase (AChE) inhibitors and memantine and reassess

6. A 72-year-old woman presented with a 10-month history of memory complaints and difficulty concentrating. Her husband described her as being more forgetful, and missing items when grocery shopping. On questioning, she described feeling tired, having trouble staying asleep, and losing 4 pounds in the last 3 months. There are no other behavioral abnormalities. She has history of hypertension and dyslipidemia. On the MMSE and MoCA scores were 27/30 and 26/30 (losing points for delayed recall), respectively. The rest of the neurological exam is otherwise normal. Blood work for reversible causes of cognitive impairment and CT head were non-contributory. Inflammatory markers and CT abdomen and pelvis were normal (no malignancy).

What would you do? Please select <u>one option from the screen</u>:

- Order brain MRI imaging and reassess
- Order Amyloid β and Tau biomarkers and reassess
- Initiate an SSRI and reassess
- Initiate a brain enhancer (e.g., Donepezil, Galantamine, etc.) and reassess
- A and B and reassess
- Refer to a geriatrician or a general neurologist (Waiting time 2-3 months)
- Refer to behavioral neurologist) (waiting time 6-9 months)
- Refer to geriatric-psychiatrist specialist (waiting time 6-9 months)
- This patient needs a follow-up to repeat the cognitive assessment before deciding if a referral or treatment are warranted.

7. A 78-year-old man came to your office for a follow-up appointment. He was diagnosed with Alzheimer Dementia (AD) 7 years ago. He has been taking Donepezil 10 mg PO daily without side effects. There have not been motor fluctuations nor behavioral abnormalities, other than anxiety. He currently experiences difficulty identifying family and friends, but still recognizes familiar faces. He misplaces objects, has become more apprehensive, and requires assistance for bathing and toileting.
He is at a moderate stage of AD with an MMSE of 17 (previous year: MSSE of 18). The rest of the neurological exam is otherwise normal. Blood work revealed mild anemia, but otherwise is non-contributory. The family would like to discuss recent treatment options given the lack of improvement with donepezil. They heard about aducanumab being approved for all stages of dementia by the FDA. They would like to know your opinion as they can get it from the USA.

What would you do? Please select <u>one option from the screen</u>:

- Continue on donepezil and refer to a general neurologist or geriatric specialist
- Order a amyloid PET scan and discuss the results with the family (as part of the eligibility criteria)
- Order Amyloid β and Tau biomarkers and discuss the results with the family
- Continue on donepezil and refer to a specialist
- Add memantine to the regimen and follow up with a cognitive assessment in 3-6 months
- Add memantine to the regimen and refer to a general neurologist
- Discuss with the family the current evidence and continue the same treatment

8. A 71-year-old accountant presented for evaluation at your office for memory and orientation problems. He believes his symptoms started 3 years ago at the time he lost his brother. Initial symptoms included mood and behavioral changes, and then word substitution emerged. His past history includes dyslipidemia, and he takes Lipitor 40 mg. There is no family history of AD or other neurological conditions. Blood work was normal. His score on the Mini-Mental State Examination (MMSE) was 27/30, MoCA score of 26 and depression was ruled out. Neuroimaging studies (MRI) revealed mild microangiopathic changes and mild atrophy.

What would you do? Please select <u>one option from the screen</u>:

- Request formal Neuropsychological assessment
- Request Amyloid β and Tau biomarker (CSF testing) before treatment
- Request amyloid PET scan before treatment
- Start treatment with acetylcholinesterase (AChE) inhibitors
- Refer to a general neurologist or geriatrician
- Reassess in 3-6 months

9. A 84-year-old woman is referred to you for assessment of memory impairment and a functional decline. She attends in the company of her son and daughter-in-law. On the pre-clinic questionnaire her son has reported a decline in her cognition (e.g., memory, language, attention span) and feeling moody or withdrawn in social events over the past 12 months. The patient herself acknowledges that there have been memory problems, but feels it is just her short-term memory that is an issue. She has history of hypertension and dyslipidemia. She scores 18/30 on the MMSE. The rest of the physical examination is otherwise normal.

What would you do? Please select <u>one option from the screen</u>:

- o Order labs to rule out reversible causes of cognitive impairment and reassess
- o Request brain imaging (MRI) and reassess
- o Request amyloid and tau biomarker (CSF testing) and reassess
- o Start an acetylcholinesterase inhibitor
- o Refer to a general neurologist or geriatrician
- o A and B would be the best course of action
- o A, B and D would be the best course of action

10. An 80-year-old man has a two-year history of gradually progressing short-term memory impairment. His family reported that he used to be sharp but over the past two years, he has demonstrated difficulty learning and remembering short-term information. He now frequently repeats questions, forgets conversations, misplaces items, and forgets having seen movies. For the last 3 months he has had tremor in the upper extremities, gait instability, and episodes of visual hallucinations of robbers in his room. His MMSE is 26/30.

What would you do? Please select <u>one option from the screen</u>:

1) Request formal Neuropsychological exam
2) Start treatment with acetylcholinesterase (AChE) inhibitors (e.g., rivastigmine)
3) Order amyloid/tau biomarker tests (CSF testing) prior to initiating treatment
4) Request functional brain imaging (FDG-PET, SPECT) before proceeding with further investigations
5) Request brain MRI and reassess
6) Refer to a geriatrician or general neurologist
7) Refer to a specialist (e.g. behavioral neurologist or geriatric psychiatrist)
8) Start treatment with an acetylcholinesterase (AChE) inhibitor (e.g., rivastigmine) and risperidone 0.5 mg HS.
9) Start treatment with an acetylcholinesterase (AChE) inhibitor (e.g., rivastigmine) and quetiapine 12.5 mg BID

--- END ---
